# Optimizing Plant Disease Management in Agricultural Ecosystems Through Rational In-Crop Diversification

**DOI:** 10.3389/fpls.2021.767209

**Published:** 2021-12-24

**Authors:** Yan-Ping Wang, Zhe-Chao Pan, Li-Na Yang, Jeremy J. Burdon, Hanna Friberg, Qi-jun Sui, Jiasui Zhan

**Affiliations:** ^1^College of Chemistry and Life Sciences, Sichuan Provincial Key Laboratory for Development and Utilization of Characteristic Horticultural Biological Resources, Chengdu Normal University, Chengdu, China; ^2^Industrial Crops Research Institute, Yunnan Academy of Agricultural Sciences, Kunming, China; ^3^Institute of Oceanography, Minjiang University, Fuzhou, China; ^4^Retired, Wright, ACT, Australia; ^5^Department of Forest Mycology and Plant Pathology, Swedish University of Agricultural Sciences, Uppsala, Sweden

**Keywords:** *Phytophthora infestans*, AUDPC, disease resistance, host mixtures, disease mitigation, agriculture sustainability, ecological disease management, multiple regression

## Abstract

Biodiversity plays multifaceted roles in societal development and ecological sustainability. In agricultural ecosystems, using biodiversity to mitigate plant diseases has received renewed attention in recent years but our knowledge of the best ways of using biodiversity to control plant diseases is still incomplete. In term of in-crop diversification, it is not clear how genetic diversity *per se* in host populations interacts with identifiable resistance and other functional traits of component genotypes to mitigate disease epidemics and what is the best way of structuring mixture populations. In this study, we created a series of host populations by mixing different numbers of potato varieties showing different late blight resistance levels in different proportions. The amount of naturally occurring late blight disease in the mixture populations was recorded weekly during the potato growing seasons. The percentage of disease reduction (PDR) in the mixture populations was calculated by comparing their observed late blight levels relative to that expected when they were planted in pure stands. We found that PDR in the mixtures increased as the number of varieties and the difference in host resistance (DHR) between the component varieties increased. However, the level of host resistance in the potato varieties had little impact on PDR. In mixtures involving two varieties, the optimum proportion of component varieties for the best PDR depended on their DHR, with an increasing skewness to one of the component varieties as the DHR between the component varieties increased. These results indicate that mixing crop varieties can significantly reduce disease epidemics in the field. To achieve the best disease mitigation, growers should include as many varieties as possible in mixtures or, if only two component mixtures are possible, increase DHR among the component varieties.

## Introduction

Agriculture is under considerable pressure to improve productivity in the face of changing environmental circumstances and a continuing rise in the earth’s human population. Plant diseases induced by pathogenic microbes cause huge annual losses that are estimated to range between 13 (Oerke, 2006) and 22% among the world’s most important food species alone, i.e., wheat, corn, soybean, potato, rice (Savary et al., 2019). This toll greatly threatens the sustainable supply of quality food, adding to concerns around food security, especially given the potential for ongoing climate change to generate conditions that may foster the geographic spread and local increase of diseases (Burdon and Zhan, 2020).

Plant diseases in agricultural ecosystems are mainly controlled by using host resistance and agrochemicals (Borrelli et al., 2018; Burdon et al., 2020; Burdon and Zhan, 2020). In the gene-for-gene system involving an effector and receptor interaction governed by major gene inheritance, resistance is often ephemeral due to rapid evolution of pathogen infectivity (Barrett et al., 2009). In contrast, quantitative resistance is polygenic and therefore more durable, but its application in disease management is threatened by the emergence of pathogen populations with higher aggressiveness and tolerance to environmental stresses (Yang et al., 2013; Zhan et al., 2015; Burdon et al., 2016). When host resistance is not available, agrochemicals become another major control option and have been widely used to produce immediate mitigation effects on pathogens either by killing them or constraining their growth and reproduction. However, this management approach is no panacea because chemicals often have detrimental effects on non-target organisms (Belsky and Joshi, 2020; Alengebawy et al., 2021) and are subject to erosion of their efficacy (Ishii and Holloman, 2015).

With increasing concern over the need for natural resource conservation (including resistance genes) and environmental wellbeing, the use of eco-evolutionary approaches to control plant diseases in agricultural ecosystems has received renewed attention in recent years (Zhan et al., 2014, 2015; He et al., 2016). One eco-evolutionary approach is to use biodiversity that, in its simplest form, utilizes mixtures of genotypes of the same species. In term of plant disease management, theoretical and empirical data suggest that increased biodiversity not only reduce plant disease epidemics in the short term but also reduce pathogen evolution in the longer term (Mundt, 2002; Zhan et al., 2002; Keesing et al., 2010; Sommerhalder et al., 2011; Burdon et al., 2016; Rimbaud et al., 2021). In addition to the suppressing effects of diversifying selection on the evolution of pathogen infectivity (Marshall et al., 2009), and of the inoculum reduction consequences caused by physical barriers that hamper pathogen dispersal, increasing biodiversity may generate indirect effects that are favourable for host immunity development but suboptimal for the survival, reproduction and transmission of pathogens (Eisenhauer et al., 2018).

Although the potential benefits of biodiversity have been identified for many decades, crop diversification has gradually reduced due to agricultural (de Lima et al., 2020) intensification and mechanization approaches that require functional uniformity of agronomic traits. However, this trend can be reversed in several ways through spatial and temporal deployment of crops by rotation, intercropping, or the use of carefully structured varietal mixtures (Kovács-Hostyánszki et al., 2017). Among these options, varietal mixtures composed of component lines with very similar agronomic traits but different genetic backgrounds and/or functional traits could be the best immediate option of applying biodiversity to control plant diseases (Zhu et al., 2000). Varietal mixtures can often be relatively easily adopted and implemented by both industrial and smallholder farmers. In contrast, other types of crop diversification such as intercropping, are often faced with agronomic, environmental, financial and other practical problems related to planting, harvesting and marketing as well as additional investments in new machines and knowledge required for the cultivation of the alternative crops (Wuest et al., 2021). The availability of technology to breed crop varieties with similar agronomic characteristics such as plant height, maturity and marketing quality but differing in other functional traits increases the potential to use varietal mixtures in sustainable commercial production (Zhan et al., 2015).

However, knowledge of the ways in which in-crop diversification can contribute to the control of plant diseases is still incomplete. For example, how much of the disease reduction in the mixtures is attributable to genetic diversity *per se* rather than to the presence of identifiable resistance genes is unclear (Schuldt et al., 2019; Rohr et al., 2020). Similarly, potential background effects of component varieties on disease reduction are yet to be clarified, as is the contribution of interactions among functional traits in the component varieties. To optimize the benefits of using in-crop biodiversity in controlling plant disease further detailed experimentation is required.

In this study, we used the potato-*Phytophthora infestans* interaction as a model system to address some of the key remaining questions associated with the contribution of in-crop diversification to plant disease management. *Phytophthora infestans* is the causal agent of potato and tomato late blights and one of the major factors limiting sustainable potato production globally. The pathogen was responsible for the Irish famine in 1840s and is still one of the main diseases in agriculture (Dean et al., 2012). It can cause up to 80% yield loss with the direct globally economic cost estimated to be >8 billion US dollars annually (Haverkort et al., 2009). In addition, billions of US dollars are spent annually on control practices including plant breeding, clean “seed” production and fungicide sprays. Previously, we combined field experiments and laboratory analyses to understand how genetic diversity in potato host populations affects potato yield, epidemics of late blight disease, the evolution of *P. infestans*, and soil fertility (Yang et al., 2019, 2021). We found that increasing genetic diversity through varietal mixtures increased potato yield and its stability, reduced late blight disease, increased microbial diversity and nutrition availability in the rhizosphere, and reduced fungicide resistance, aggressiveness, and the evolutionary rate of the pathogen. Here, we reanalyse some of these published data together with additional data generated from a new experiment. Our aim is to understand the interaction of genetic diversity with other functional traits such as host resistance and how this interaction affects disease epidemics. Such information is important in optimizing the effectiveness of plant disease management through the rational deployment of available varieties.

The specific objectives of the current study were to:

(1) Understand the quantitative contribution of genetic diversity in potato populations to late blight epidemics;
(2) Evaluate the relative impact of the level and difference in resistance in component potato varieties on late blight epidemics; and
(3) Optimize the proportions of component varieties for best late blight control.

## Materials and Methods

### Field Experiments

Two experiments were conducted in Yunnan, southwest China using a series of potato populations varying in genetic diversity and host resistance. Yunnan is a main potato production region of China, accounting for ∼10% of total acreage (Liao and Guo, 2014). It also represents one of the most favourable ecosystems for potato production and late blight disease epidemics. Potato can be grown year-round in the region and late blight is the major constraint on production. Host diversification primarily through intercropping has been widely used in the region to control plant diseases (Zhu et al., 2000; Luo et al., 2021) including potato late blight (Yang et al., 2019). Our experimental populations were created by planting potato either in mixtures of various number of varieties or in pure stands. The varieties used in these experiments shared tuber colour, plant height and maturity, thereby minimizing any obvious differences that might affect their management in the field or marketing value.

Experiment 1 was conducted in two fields located at Yema (26.10° N, 103.38° E) and Xiaoshao (25.16° N, 102.00° E) in 2012 and 2013, respectively. Details of the experimental design can be found in a previous publication (Yang et al., 2019). Briefly, six potato varieties differing in functional traits such as *P. infestans* resistance (ranging from highly resistant to highly susceptible) but similar in maturity and plant height were planted in pure stands or in random mixtures of equal proportions of two, three, four, five or six varieties, respectively. With six potato varieties, a total of 63 potential combinations/populations can be formed (Table 1). Due to resource constraints, only 31 treatments, composed of six each of the pure stands, two-, three-, four-, and five-variety mixtures and one of the six-variety mixture, were planted using a Random Completely Block design (RCBD) with three replicates. To prevent potential confounding contributions of varietal background to the effect of host genetic diversity on late blight disease epidemics, the 31 host combinations/populations were formed in such a way that all varieties were equally represented in each category of genetic diversity (i.e., the 6 varieties are equally represented across each of the mixture groupings). For example, among the 3-varietal mixtures, each variety appears in three different mixture combinations (Table 1).

**TABLE 1:** The composition of potato populations tested in the field experiment and the proportion of potato varieties in each of the diversity levels.

Diversity level	Host treatments (populations)			
	Potential	Tested	Composition of the tested populations	PVCDL^2^
1	6	6	V^1^1, V2, V3, V4, V5, V6	1/6
2	15	6	V1V4, V1V6, V2V5, V2V6, V3V4, V3V5	1/6
3	20	6	V1V2V6, V1V3V6, V1V4V6, V2V3V5, V2V4V5, V3V4V5	1/6
4	15	6	V1V2V4V5, V1V2V4V6, V1V3V4V6, V1V3V5V6, V2V3V4V5, V2V3V5V6	1/6
5	6	6	V1V2V3V4V5, V1V2V3V4V6, V1V2V3V5V6, V1V2V4V5V6, V1V3V4V5V6, V2V3V4V5V6	1/6
6	1	1	V1V2V3V4V5V6	1/6

*^1^V, variety;*

*^2^PVCDL, proportion of each variety in corresponding diversity group.*

Experiment 2 was conducted only at Yema during the 2015 and 2016 potato growing seasons. In this experiment, four potato varieties different to those used in Experiment 1 were divided into two groups according to their level of resistance to late blight. The two varieties (A & B) in one group, denoted as Group S (susceptible), were susceptible to potato late blight although variety A was more susceptible than variety B as measured by the amount of disease suffered under field epidemic conditions using the area under the disease progress curve (AUDPC, Table 2). In the second group, denoted as Group MR (moderate resistance), the two varieties were almost identical in their level of resistance to late blight. In each group, the two varieties were planted either as pure stands or as a series of 9 mixtures differing by 10% in relative proportions (i.e., 10/90%, 20/80%, 30/70%, 40/60%, 50/50%, 60/40%, 70/30%, 80/20%, 90/10%). The field experiment was laid out in a split-plot design with “group” in the main plot and “treatment” in the split plot. The experimental plots were replicated three times.

**TABLE 2:** Multiple regression analyses to determine the relative contribution of host resistance level (RES) estimated by AUDPC when the varieties were grown in pure stands, difference in host resistance (DHR) between the component varieties, and host diversity (HDV), which was measured by the number of varieties grown together in a field treatment to the percentage of late blight reduction in potato mixtures.

	Coefficients	Lower 95%	Upper 95%	*P*-value
RES	−1.4E-05	−9.1E-05	6.36E-05	0.726951
Ln (HDV)	**0.397955**	0.266601	0.52931	5.99E-09
RES × Ln (HDV)	−**0.0002**	−0.00029	−0.00011	1.61E-05

RES	0.000271	−5.4E-05	0.000597	0.101037
DHR	**1.367027**	0.64035	2.093705	0.000363
RES × DHR	−**0.00065**	−0.00111	−0.00018	0.007087

*Note: Values with bold font are significant at p < 0.01.*

Each plot was composed of six rows with a total area of ∼25 m^2^ in both experiments. The fields were managed in accord with local commercial production practices. In both experiments, disease epidemics were initiated by natural infection of the pathogen over-seasoning on potato debris and/or secondary hosts in the local or surrounding area. In this region of Yunnan, late blight disease occurs almost every year and the pathogen is genetically and phenotypically highly variable, for example, in race complexity (Wu et al., 2016), pathogenicity (Zhu et al., 2016) and stress tolerance (Wu et al., 2018), ensuring the successful establishment of the disease in our experiments. Late blight severity was estimated visually and recorded weekly in each field plot from the emergence of potato plants to the end of the growing season.

### Statistical Analysis

Total late blight disease levels in each plot was determined by AUDPC (Fry, 1978) calculated from disease severity quantified weekly over the growing season. The expected disease severity (EDS) in the mixtures was estimated according to the proportion of each variety in the mixture and the observed disease severity (ODS) when grown in pure stand as follows:


$$\text{EDS}=\sum_{i}\left(P_{i}\,\text{x}\,O\,D\,S_{i}\right)$$

Where *P*_*i*_ and ODS_i_ refer to the proportion and observed disease severity in variety *i* (*i* = 1 to 6), respectively.

Host resistance in the varieties was estimated by AUDPC when they were grown in pure stands. Difference in host resistance (DHR) between varieties, which was only calculated for the two-variety mixtures from Experiment 1, was estimated using the following formula:


$$\text{DHR}=\frac{\left|O\,D\,S_{1}-O\,D\,S_{2}\right|}{O\,D\,S_{1}+O\,D\,S_{2}}\,\mathrm{x100}\%$$

Where ODS_1_ and ODS_2_ are the observed disease severities of the two-component varieties when they were grown in pure stands.

PDR (percentage of disease reduction) in the mixtures was estimated using ODS and EDS as follow:


$$\text{PDR}=\frac{\mathrm{ODS}-\mathrm{EDS}}{\text{EDS}}\,\text{x}\,100\%$$

Analysis of variance (ANOVA) was performed by a general linear model using the SAS programme. In this analysis, potato diversity and host resistance were treated as random variables and proportion as a fixed variable. Least significant difference was used to compare disease severity and/or PDR among treatments. Disease severity and PDR data were fitted to statistical models and evaluated through the Akaike information criterion (AIC, Sakamoto et al., 1986) if more than one model was statistically supported. In the evaluation, models with a smaller AIC value are better but were not considered to be significantly so if the difference of AICs between models was less than two units (Zhan et al., 2021). For data fitted to a quadratic distribution, their modes were estimated and used to determine the optimal mixtures for the best late blight management. A two-tailed *T*-test was used to compare the modes generated from different treatment groups, i.e., S and MR groups. Multiple regression fitting was also used to determine the relative contribution of host resistance, DHR and host genetic diversity *per se* (measured by the number of varieties grown together in a field treatment), to disease development in the mixtures.

## Results

**FIGURE 1:**
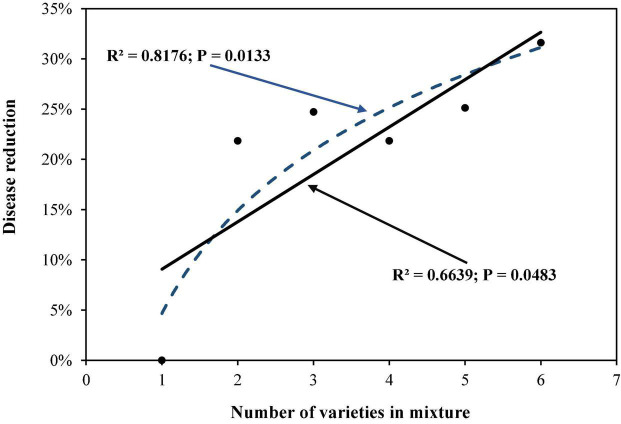
The effect of varietal number in the mixtures on potato late blight disease caused by *Phytophthora infestans.*

Analysis of variance revealed that the number of varieties, level of host resistance, DHR, and varietal proportion in the mixtures all had a significant impact on the total late blight and/or PDR (*P* < 0.01). Further assessment using partial correlation analysis indicated that PDR increased as the number of varieties in the mixtures increased (Figure 1) with the logarithmic model outperforming the linear model (Figure 1) as indicated by the AIC test. In line with predictions of the better fitting logarithmic model, PDR increased more rapidly as the first few varieties were added relative to levels predicted under the linear model. As the number of varieties continued to increase, PDR started to slowly flatten out (Figure 1). Although the partial correlation analysis also pointed to a linear association between PDR and the level of host resistance, this effect was caused by the interaction between host resistance and genetic diversity as shown by the multiple regression analysis (Table 2).

**FIGURE 2:**
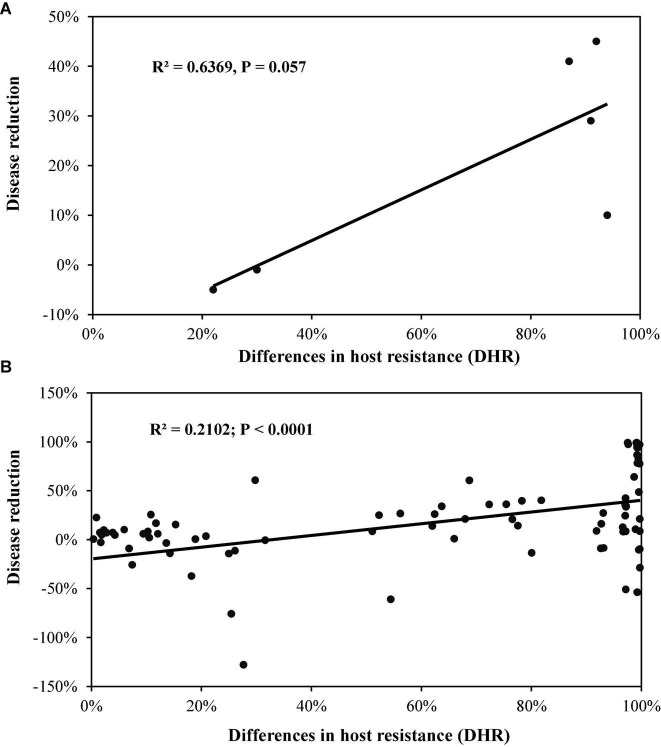
The effect of difference in host resistance (DHR) between the component varieties in the two varietal mixtures on disease reduction of potato late blight caused by *Phytophthora infestans*: **(A)** Means estimated from fields across replicates, locations, and years of Experiment 1; **(B)** individual plot scores in each replicate, year and location of Experimental 1.

When data from the two-component mixtures in Experiment 1 was analysed separately, PDR increased linearly as DHR increased, but this association was only marginally significant (Figure 2A). The association became highly significant when data points were increased by considering late blight and host resistance according to each replicate, location, and/or year (Figure 2B; Supplementary Figure 1). Multiple regression analysis from this part of the data also indicated that host resistance did not affect PDR (Table 2).

**FIGURE 3:**
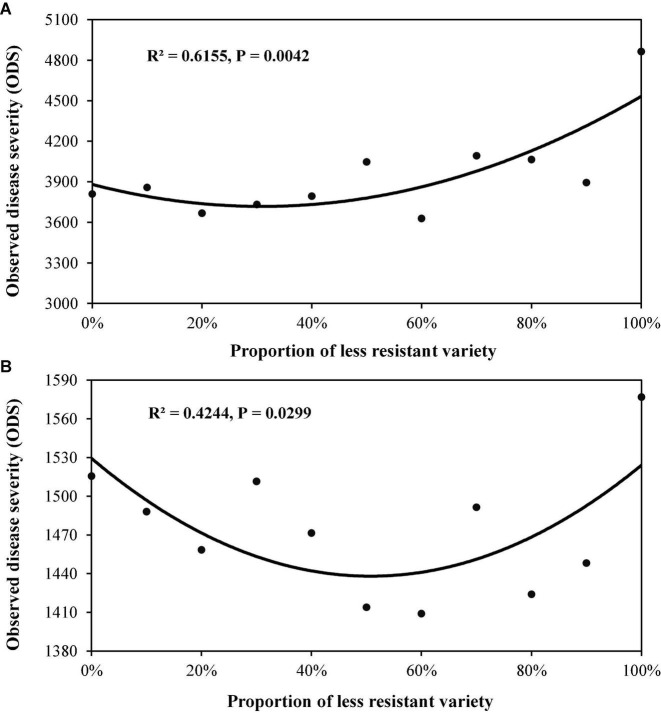
Cumulative potato late blight disease measured by AUDPC for various proportions of two-variety mixtures. The AUDPC was calculated from observed disease severity over the entire potato growing season: **(A)** the mixture of two varieties (Group S) with lower disease resistance (higher AUDPC value); and **(B)** the mixture of two varieties (Group MR) with higher disease resistance (lower AUDPC value).

**FIGURE 4:**
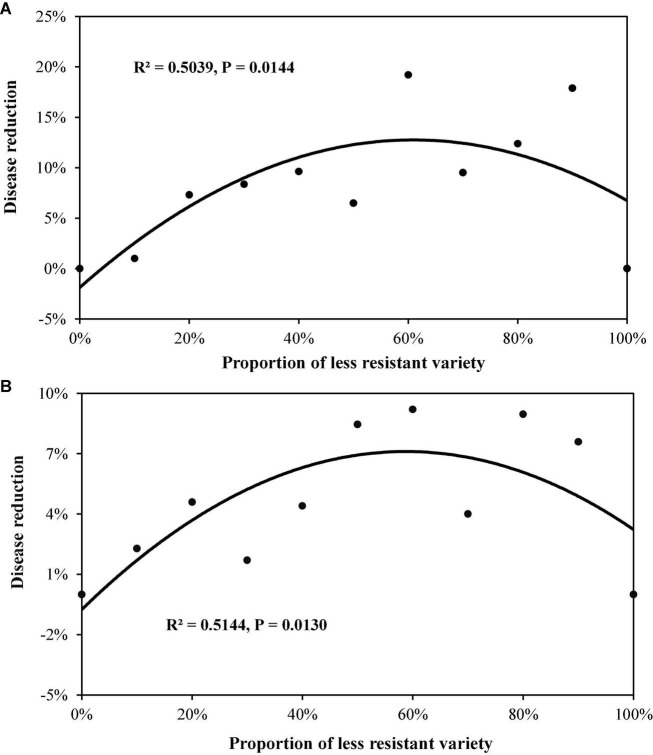
The extent of potato late blight disease reduction under different proportions of two-variety mixtures. Disease reduction was estimated by weighting the difference between observed and expected disease severity with the expected disease severity: **(A)** the mixture of two varieties (Group S) with lower disease resistance (higher AUDPC value); and **(B)** the mixture of two varieties (Group MR) with higher disease resistance (lower AUDPC value).

In Experiment 2, all two-variety mixtures reduced late blight disease relative to the mean of pure stands regardless of host resistant level and DHR (Figures 3, 4). An average of 10.2% disease reduction with a mode of 14.3% (expected maximum reduction) was observed in Group S in which the average AUDPC in the two varieties when they were grown in pure stands was 4,337 (lower host resistance). In contrast, only an average of 5.7% disease reduction, with a mode of 7.0%, was found in Group MR in which the average AUDPC of the two varieties when they were planted in pure stands was 1,547 (higher host resistance, Table 3). Both the observed PDR and its expected maximum (mode) in the Group S were significantly higher than those in the Group MR. Both ODS and PDR fitted quadratic distributions well (Figures 3, 4). In Group MR where the level of resistance in the two varieties was the same, the mode of the quadratic distribution was found to be located at the point where two varieties were planted in the proportions of 51:49 (Figure 3A; Table 3), indicating that the least amount of late blight disease would occur if the two varieties were planted in equal proportions. In contrast, in Group S where the AUDPC ratio of varieties was 56:44, the mode of the quadratic distribution was located at the point where two varieties were planted in a 69:31 ratio (Figure 3A; Table 3), indicating the least disease occurrence would be achieved only when the more susceptible variety was planted in a lower proportion. However, in term of PDR, the best results were achieved by planting more susceptible varieties (∼60%) in both situations (Figure 4).

**TABLE 3:** Estimates of optimal varietal compositions and their corresponding efficacy in mitigating late blight disease in the two mixtures varying in host resistance measured by AUDPC and in difference of host resistance between component varieties.

Treatments	AUDPC in pure stand			Optimal B (%)		Disease reduction (%)	
	Variety A	Variety B	B : A	Least disease	Best disease reduction	Mean Obs.	Exp. Max (mode)
Group S	3,809 (±553)	4,865 (±637)	56:44	31:69	61:39	10.2 A	14.3 A
Group MR	1,516 (±54)	1,577 (±50)	51:49	51:49	59:41	5.7 B	7.0 B

## Discussion

Increasing host diversity by growing different genotypes of the same species together has been proposed as an environmentally friendly and economically justifiable approach to plant disease management, and many studies have been conducted to understand the mechanisms behind this strategy. However, results from empirical studies are not always consistent. While some have shown that diversity enrichment in host populations makes a positive contribution to crop yield and disease management, other studies have failed to achieve the expectation (Ganz and Ebert, 2010; Gibson and Nguyen, 2021). This inconsistency suggests that the efficacy of using in-crop diversity to reduce disease may be a variety-dependent phenomenon, possibly attributable to eco-evolutionary differences associated with functional characteristics of varieties. Indeed, it is hypothesised that varietal mixtures are only effective in delivering disease reductions when there is DHR among varietal components (Xu, 2012). Our results partially falsify this hypothesis. We found that mixing two varieties with the same level of host resistance also significantly reduced the amount of disease in fields although PDR was more apparent as DHR between the varietal components increased (Figures 2–4). However, the level of host resistance *per se* does not have a direct impact on late blight epidemics. Rather, its contribution to PDR in the mixture is indirect through its interaction with other functional traits such as host diversity and DHR among varietal components. These phenomena are likely caused by fitness penalties that occur when allo-deposition events involving cross-transmission of pathogens from different host varieties result in reductions of infection or lesion development (Marshall et al., 2009). As DHR between the two host varieties in the mixture increases, this cross-transmission fitness penalty also increases, leading to greater PDR. These results suggest that in practices involving only two-variety mixtures, we should aim to maximize DHR to increase disease reduction efficiency. For example, if there are two groups of varieties with one group being disease resistance while another group is disease susceptible, we should mix varieties from different rather the same group to achieve best disease control. However, a two-component mixture approach is still effective in reducing disease epidemics even if there is no possibility of mixing varieties with sufficient DHR due to inappropriate combinations of other agronomical traits such as plant height, harvest time, yield, or consumer preference. These agronomical traits should be as identical as possible for both practical and marketing reasons, and should be of primary consideration in selecting varieties for mixtures.

In this study, we move beyond the traditional focus on two-component to multiple-component mixtures including up to six different varieties. This design allowed us to directly quantify the efficacy of using genetic diversity to control plant disease by conducting a correlation analysis between disease seen in the field and the number of varieties involved in the mixtures. We used two approaches to remove variety-dependent phenomena directly by creating potato populations in such a way that the contribution of the genetic background from individual varieties was equally weighted and indirectly by partitioning disease variance to different factors (Table 2). Similar to the previous result estimated from observed disease severity (Yang et al., 2019), our analysis indicates that PDR also increases in response to an increase in varietal number included in the mixtures even though this additional gain in disease control efficacy declines and eventually may asymptote to a constant maximum as further varieties are added (Figure 1). In addition to a fitness penalty associated with allo-deposition events (Schoeny et al., 2008), increasing travel distance of pathogens (Madden, 1997) and a diluting effect on inoculum density (Haas et al., 2011), other eco-evolutionary elements may also contribute to the observed result. For example, balanced uptake of fertilisers applied and diversified secretion of compounds required for mineral element release from soils by different host varieties increases nutrition availability and efficiency for healthy crops (Zak et al., 2003). Increasing genetic diversity of microbial communities in rhizosphere by host varietal diversification can serve both as a promoter of fossil element release and competitor to reduce the reproduction and transmission of pathogens (Lambers et al., 2009). These positive effects of biodiversity on host health and negative effects on pathogen survival and transmission create a suboptimal environment for disease epidemics in the short term and pathogen evolution in the long term. These ecological phenomena have been documented widely in both agricultural and natural ecosystems (Finckh et al., 2000; Loreau et al., 2001).

To maximise the effect of variety mixture on disease reduction, the proportion of different varieties in the mixture populations should be carefully adjusted (Garrett and Mundt, 1999). Our results indicate that the best disease reduction is not always associated with equal mixture of varietal components that provides the expected highest diversity in host populations (Nei, 1973). To achieve the lowest total disease severity (Figure 3), the relative abundance of the two varieties is roughly reflected by their DHR with a skewness toward the variety with the higher host resistance (Table 3). For example, if total disease reduction is the primary consideration and the ratio of host susceptibility in the higher and lower susceptible variety is 70:30, then the lowest total disease in the field occurs when the two varieties are planted at the ratio of 30:70. On the other hand, if there is a preference for the more susceptible variety due to its yield potential or quality, etc., it can be planted at greater than 50% in the mixture as indicated by the continuing increase in PDR as the proportion of more susceptible variety in the mixture increased (Figure 4). However, our results were only derived from two pairs of treatments generated from mixture of varieties with the same resistant level. It is not clear whether our observation is a specific case to our experiments or is the general phenomenon in the agricultural systems. Further research should be conducted with more pairs of mixtures including mixture of varieties among resistant groups to confirm this result.

In agriculture, crop varieties favoured by farmers are often associated with higher productivity or good quality but lower disease resistance (Vyska et al., 2016). Due to their susceptibility, these varieties have to be withdrawn from commercial use or rely on heavy application of agrichemicals to ensure necessary levels of productivity – a production strategy that is often associated with enhancing evolutionary changes in the pathogen, deleterious effects on the environment and increasing economic costs for growers and society as a whole (Lechenet et al., 2017; He et al., 2021; Wright et al., 2021). In developing countries where smallholder farmers lack the resources for extensive purchase of agrichemicals, these financial burdens are a particular concern. The breeding of replacement varieties with similar production potential, quality and consumer acceptance is time consuming and requires additional genetic and financial resources. Our research provides empirical guidance on how to effectively extend the useful life of crop varieties whose resistance has been overcome to ensure sustainable agricultural production without additional financial or environmental burdens. If many varieties are available for use, a complex mixture involved several varieties (≥5) should be considered to increase the efficacy of disease reduction. However, if the number of varieties available are limited, the simplest mixture of two varieties could also achieve the goal of disease reduction. In this case, choice should go to varieties with levels of host resistance that are as different as possible.

## Conclusion

Our results indicate that mixing crop varieties can significantly reduce disease epidemics in the field, but planting varieties in equal proportion, as it is commonly practised, does not necessarily generate the best disease control. To achieve the best disease mitigation, growers should include as many varieties as possible in mixtures or, if only two component mixtures are possible, increase DHR (difference in host resistance) among those component varieties. In the latter case, the percentage representation of the component varieties should be carefully adjusted to reflect their DHR. The more susceptible variety should be less represented in the two-component mixtures if disease control is the primary consideration but can be planted at a higher proportion if the variety is preferred by farmers due to its quality or yield potential.

## Data Availability Statement

The raw data supporting the conclusions of this article will be made available by the authors, without undue reservation.

## Author Contributions

Y-PW and Z-CP collected and analyzed data and wrote the manuscript. L-NY collected and analyzed data. JB and HF wrote the manuscript. Q-JS conceived the project and managed the field experiment. JZ conceived, designed and supervised the experiments analyzed the data, and wrote the manuscript. All authors reviewed the manuscript.

## Conflict of Interest

The authors declare that the research was conducted in the absence of any commercial or financial relationships that could be construed as a potential conflict of interest.

## Publisher’s Note

All claims expressed in this article are solely those of the authors and do not necessarily represent those of their affiliated organizations, or those of the publisher, the editors and the reviewers. Any product that may be evaluated in this article, or claim that may be made by its manufacturer, is not guaranteed or endorsed by the publisher.

## Supplementary Material

Supplementary Figure 1 The effect of difference in host resistance between the component varieties in the two varietal mixtures on potato late blight reduction caused by *Phytophthora infestans*: **(A)** Individual plot scores in 2012; **(B)** Individual plot scores in 2013; **(C)** Individual plot scores in Xiaoshao; and **(D)** in Yema.
