# Supplementary figures and images for: Optimizing Plant Disease Management in Agricultural Ecosystems Through Rational In-Crop Diversification
Source: Front Plant Sci. 2021 Dec 24;12:767209. doi: 10.3389/fpls.2021.767209 (PMC8739928; doi:10.3389/fpls.2021.767209)

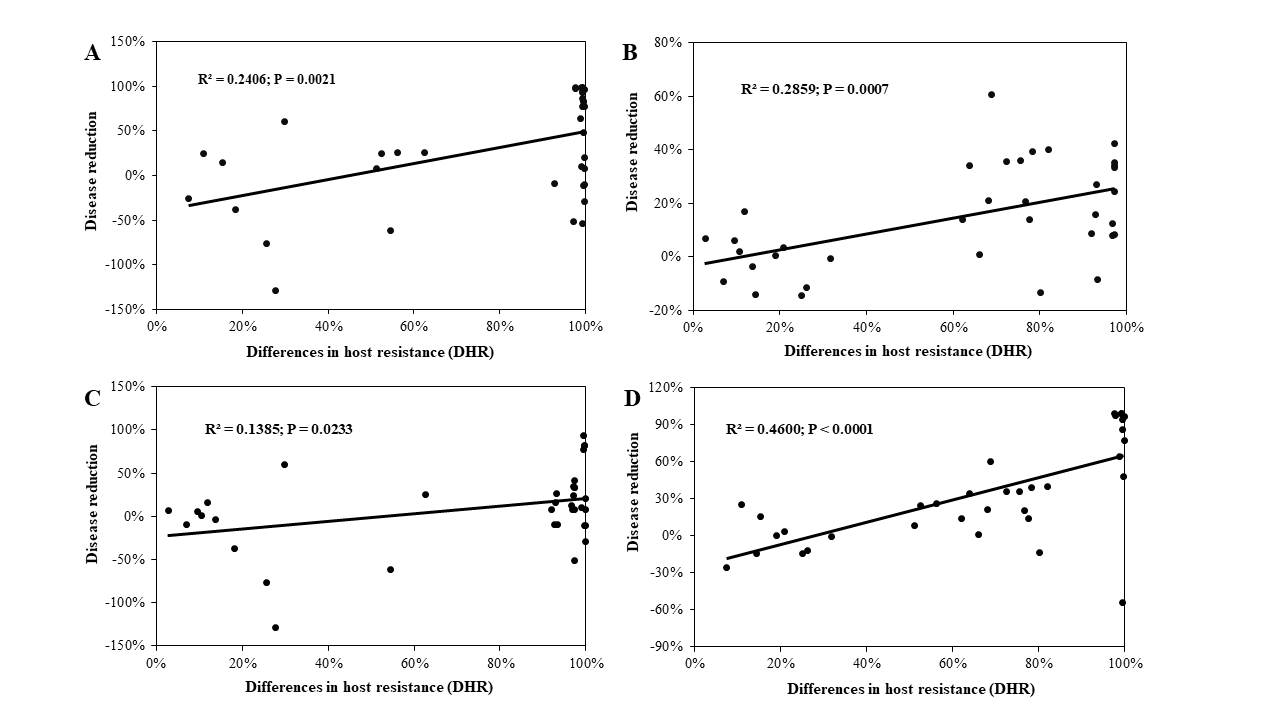

Supplement: Supplementary Figure 1 — The effect of difference in host resistance between the component varieties in the two varietal mixtures on potato late blight reduction caused by Phytophthora infestans: (A) Individual plot scores in 2012; (B) Individual plot scores in 2013; (C) Individual plot scores in Xiaoshao; and (D) in Yema. [file Image_1.tif]
